# Effect of Cultural Adaptation of a Smartphone-Based Self-Help Programme on Its Acceptability and Efficacy: Randomized Controlled Trial

**DOI:** 10.32872/cpe.2743

**Published:** 2024-06-28

**Authors:** Eva Heim, Sebastian Burchert, Mirëlinda Shala, Anna Hoxha, Marco Kaufmann, Arlinda Cerga Pashoja, Naser Morina, Michael P. Schaub, Christine Knaevelsrud, Andreas Maercker

**Affiliations:** 1 Institute of Psychology, University of Lausanne, Lausanne, Switzerland; 2 Department of Education and Psychology, Division of Clinical Psychological Intervention, Freie Universität Berlin, Berlin, Germany; 3 Department of Economics, Lucerne University of Applied Sciences and Arts, Lucerne, Switzerland; 4 Department of Psychology, University of Zurich, Zurich, Switzerland; 5 Epidemiology, Biostatistics and Prevention Institute, University of Zurich, Zurich, Switzerland; 6 Faculty of Population Health, London School of Hygiene and Tropical Medicine, London, United Kingdom; 7 St Marys University, Twickenham, London, United Kingdom; 8 Department of Consultation-Liaison Psychiatry and Psychosomatic Medicine, University Hospital of Zurich, University of Zurich, Zurich, Switzerland; 9 Swiss Research Institute for Public Health and Addiction, Zurich, Switzerland; Friedrich-Alexander-Universität Erlangen-Nürnberg, Erlangen, Germany

**Keywords:** cultural adaptation, psychological interventions, mobile mental health, self-help, immigrants, online interventions, cultural concepts of distress, fatalism, working alliance

## Abstract

**Background:**

Research on cultural adaptation of psychological interventions indicates that a higher level of adaptation is associated with a higher effect size of the intervention. However, direct comparisons of different levels of adaptations are scarce.

**Aims:**

This study used a smartphone-based self-help programme called Step-by-Step (Albanian: Hap-pas-Hapi) for the treatment of psychological distress among Albanian-speaking immigrants in Switzerland and Germany. Two levels of cultural adaptation (i.e., surface vs. deep structure adaptation) were compared. We hypothesised that the deep structure adaptation would enhance the acceptance and efficacy of the intervention.

**Method:**

We conducted a two-arm, single-blind randomised controlled trial. Inclusion criteria were good command of the Albanian language, age above 18, and elevated psychological distress (Kessler Psychological Distress Scale score above 15). Primary outcome measures were the total score of the Hopkins Symptom Checklist and the number of participants who completed at least three (out of five) sessions. Secondary outcomes were global functioning, well-being, post-traumatic stress, and self-defined problems.

**Results:**

Two-hundred-twenty-two participants were included, of which 18 (8%) completed the post-assessments. The number of participants who completed the third session was equal in both conditions, with N = 5 (5%) and N = 6 (6%) respectively.

**Discussion:**

Drop-out rates were high in both conditions, and no group difference was found regarding the acceptance of the intervention. The high drop-out rate stands in contrast with other trials testing Step-by-Step. Future research should examine cultural factors impacting recruitment strategies, as insights could help to reduce participant drop-out rates in clinical trials.

## Highlights

- An online self-help intervention was adapted to Albanian’s cultural concepts of distress.
- In a randomised controlled trial, two levels of cultural adaptation were compared.
- Recruitment was slow, and drop-out rates were high (92%).
- No difference was found between the two levels of adaptation with regard to acceptance.

## Background

Common mental disorders (CMDs) such as depression, anxiety, and post-traumatic stress disorder (PTSD) contribute to a significant burden of disease worldwide (Whiteford et al., 2013), particularly among populations affected by armed conflicts and migrant populations (Charlson et al., 2016; Turrini et al., 2017). Negative effects of violent conflicts and migration on mental health often persist over years or decades (de Jong et al., 2003), e.g., in survivors of the Balkan wars (Bogic et al., 2015; Priebe et al., 2010). However, migrant populations often do not have access to care due to barriers such as poor command of the host country language, cultural beliefs about mental health, lack of trust towards mental health services, and mental health-related stigma (Priebe et al., 2016).

Internet-based interventions are currently propagated as one potential measure to address the worldwide mental health treatment gap (Schröder et al., 2016). Meta-analytic evidence shows that these interventions are efficacious (Andersson et al., 2019), and there is a growing number of studies testing them among culturally diverse groups (Naslund et al., 2017).

One RCT in the Netherlands examined the efficacy of a culturally adapted online-intervention for the treatment of depression among Turkish immigrants (Ünlü Ince et al., 2013). No significant difference was found in symptom improvement in the experimental group compared to the control group, although the same intervention had shown a medium effect size in a Dutch sample (van Straten et al., 2008). The drop-out rate among Turkish participants was 42% at post-test and 62% at three-months follow-up, compared to 17% at post- and follow-up in the Dutch sample. These results indicate that the efficacy and acceptance of online mental health interventions may not be the same if they are applied in a group that is ethnically or culturally different from the one it was developed for.

World Health Organization (WHO), in collaboration with the Ministry of Public Health in Lebanon, the Freie Universität (FU) Berlin, and the University of Zurich, have developed an online intervention called Step-by-Step for the treatment of depression among culturally diverse groups (Carswell et al., 2018). Step-by-Step was written in English and developed in a “generic” approach, designing illustrations and narratives in a way that they can potentially speak to people from different contexts (Carswell et al., 2018). Thereafter, it was culturally adapted for different cultural groups living in Lebanon (Abi Ramia et al., 2018). Effectiveness and cost-effectiveness of Step-by-Step was tested in two parallel RCTs among Syrian refugees and other people residing in Lebanon (*N* = 1,249 in total), showing intervention effects on depression and impaired functioning, among other outcomes (Cuijpers et al., 2022a; Cuijpers et al., 2022b). Step-by-Step is also currently being tested in three parallel RCTs among Syrian refugees in Germany, Sweden, and Egypt (*N =* 500 per site) within the EU-funded STRENGTHS project (Sijbrandij et al., 2017).

There is an ongoing debate on the extent to which cultural adaptation of psychological interventions contributes to their acceptability, efficacy, and effectiveness. Culture is related to how symptoms are expressed (Haroz et al., 2017; Kohrt et al., 2014) and how different cultural groups explain the emergence of such symptoms, which is also known as explanatory models (Bhui & Bhugra, 2002; Bhui et al., 2006). Explanatory models reveal people’s implicit assumptions about mind-body relationships and religious or spiritual beliefs (e.g., Kohrt & Hruschka, 2010). In DSM-5, culturally diverse idioms of distress and explanatory models are subsumed under the term cultural concepts of distress (CCD) (American Psychiatric Association, 2013; Kohrt et al., 2014).

Evidence on cultural adaptation of psychological interventions indicates a benefit of adapting psychological interventions to the target group (Griner & Smith, 2006; Harper Shehadeh et al., 2016; Smith et al., 2011). However, it remains unclear *what* has to be adapted, and what the benefits are (Heim & Knaevelsrud, 2021). Resnicow et al. (1999) differentiate between *surface* and *deep structure* adaptations to health interventions. Surface adaptations refer to matching materials (e.g., illustrations, language), as well as channels and settings for treatment delivery to observable characteristics of the target population. By contrast, deep structure adaptations take into account how cultural, social, environmental, or historical factors influence health behaviours. Such adaptations are based on assumptions of how members of a particular cultural group perceive the cause, course, and treatment of a particular illness, thus refer to CCDs (Heim & Kohrt, 2019). However, there is not much evidence on the effects of such adaptations. Direct comparisons of adapted and non-adapted versions of the same interventions are scarce. One meta-analysis found a medium effect size (Hedge’s *g* = .52) for such direct comparisons (Hall et al., 2016), but this was based on a small number of studies.

When adapting interventions to CCD, addressing fatalism might be one key aspect. An ethnopsychological study among Albanian-speaking immigrants in Switzerland (Shala et al., 2020b) showed that participants understood their suffering as part of normal life, given by God or fate (*fati*), and something that cannot be cured but has to be borne with endurance (*durim*). Lohaus and Schmitt (1989) differentiate between internal beliefs about health (i.e., beliefs that one can influence health or illness), social-external beliefs (i.e., beliefs that other people, including health workers, can influence health or illness), and fatalistic-external beliefs (i.e., beliefs that health and illness depend on luck or destiny). Thus, Albanian-speaking immigrants in Switzerland seem to hold fatalistic-external health beliefs. A similar concept of suffering has also been described among Turkish immigrants in Germany (Franz et al., 2007; Reich et al., 2015). Reich et al. (2021) developed a web-based intervention to enhance motivation for psychotherapy among Turkish immigrants in Germany. In a pilot study, they found that this intervention enhanced treatment motivation and reduced fatalistic-external beliefs. Fatalism might therefore be a relevant aspect in cultural adaptation of psychological interventions for migrants.

In the present study, we aim to compare surface vs. deep structure adaptation of the online intervention Step-by-Step (Albanian: Hap-pas-Hapi) for Albanian-speaking immigrants in Switzerland and Germany. We conducted an ethnopsychological study to examine the target group’s CCD (Shala et al., 2020b). This study revealed specific idioms of distress, which were used for the deep structure adaptation. The study also showed that the target population held fatalistic-external beliefs. A modified version of the intervention developed by Reich et al. (2021) will be used in the deep structure adaptation of Step-by-Step in the present study.

## Method

### Aims and Design

The culturally adapted, Smartphone-based self-help intervention called Hap-pas-Hapi (Albanian for Step-by-Step) for the treatment of depression was tested in a two-arm, single-blind RCT among Albanian immigrants in Switzerland and Germany. Hap-pas-Hapi starts with an introduction and then offers five sessions (see below). In this study, one group had access to the Albanian translation of Hap-pas-Hapi that only includes surface adaptations (Resnicow et al., 1999), and the other group received a version of Hap-pas-Hapi that was adapted to the target populations’ CCD (i.e., deep structure adaptation). The deep structure adaptation was done based on an ethnopsychological study conducted in the target population (Shala et al., 2020b) and is described more in detail elsewhere (Shala et al., 2020a). Based on current literature, we hypothesised higher efficacy (first primary outcome) and treatment adherence (second primary outcome) in the deep structure when compared to the surface adaptation version.

More specifically, we hypothesised that the deep structure adaptation would decrease participants’ fatalistic-external health beliefs (Reich et al., 2021) and enhance their working alliance with the programme (Gómez Penedo et al., 2020). We hypothesised that fatalistic-external beliefs would mediate the relationship between cultural adaptation and efficacy (first primary outcome), and working alliance would mediate the relationship between cultural adaptation and adherence (second primary outcome). A mediation effect can only be shown if the change in the mediator occurs before the change in symptoms (Lemmens et al., 2016). Therefore, working alliance was measured at the end of the introduction and session 1. Control beliefs and severity of symptoms were measured at baseline, before starting session 3, and at the end of the programme (for assessments and time points, see Heim et al., 2020S-b).

Finally, we aimed to test whether the required cultural adaptation of an intervention interacted with the level of acculturation among the target population. More precisely, we assumed that the less Albanian-speaking immigrants adopted the receiving (i.e., Swiss and German) culture and the more they retained their culture of origin, the higher the effect of cultural adaptation on treatment adherence and efficacy.

### Participants

Inclusion criteria were: a) Albanian-speaking, b) age 18 or above, c) a score of 15 or higher on the Kessler Psychological Distress Scale (K10, Kessler et al., 2002), Albanian version (Hyseni Duraku et al., 2018), and d) access to Internet (Smartphone or web-browser on tablet or computer). Exclusion criteria were: a) living outside Switzerland and Germany, b) serious suicidal thoughts or plans (self-assessed with a corresponding question). We did not explicitly screen for mental disorders but assumed that people with severe mental disorders (e.g., acute psychosis) would not be able to sign-up and go through the onboarding procedure. We did not exclude people due to severe mental disorders.

### Intervention

Step-by-Step is an online intervention for the treatment of depression that can be accessed through a mobile app (iOS or Android) or a web browser (Burchert et al., 2019; Carswell et al., 2018). It uses a narrative approach, in which an illustrated character tells his recovery story. An illustrated doctor narrator provides psychoeducation and interactive exercises. The therapeutic components are behavioural activation, stress management, positive self-talk, mood tracking, strengthening social support, and relapse prevention (Carswell et al., 2018).

Step-by-Step can be used as a guided or unguided mental health intervention. Guidance was provided in the Lebanon trials (Cuijpers et al., 2022a; Cuijpers et al., 2022b). So-called “e-helpers”, i.e., trained non-specialists, contacted participants weekly through phone or chat and provided minimal guidance (max. 15 minutes per week). In the present study, we used a “contact on-demand” model, in which e-helpers (, i.e., Albanian-speaking students working in our team, and trained to communicate with participants in the study) responded to users’ questions but did not proactively reach out to participants.

Step-by-Step was translated into Albanian language and adapted through two different approaches. The first, surface adaptation (Resnicow et al., 1999) was based on a “cognitive interviewing” technique, in which users read through the intervention and provide comments on the content, illustrations, exercises, and the usability of the intervention. A similar approach was used for the cultural adaptation of Step-by-Step in Lebanon (Abi Ramia et al., 2018). The deep structure adaptation was done based on an ethnopsychological study conducted in the target population (Shala et al., 2020b) and included three components: i) idioms of distress of the target population, ii) a new exercise in the introduction, in which the treatment rationale was adapted to the target populations’ explanatory models, and iii) a goal-setting task (added to the introduction, as well), which aimed to address participants’ socio-centric concept of the self (Kirmayer, 2007). The goal-setting task focused on the potential benefits of using Hap-pas-Hapi for the family or community at large. Thus, the main adaptations were done to the introduction (before starting session 1) with the aim of enhancing treatment motivation and adherence. The deep structure adaptation is described more in detail elsewhere (Shala et al., 2020a).

### Recruitment

Participants were recruited through three streams: social media (e.g., Facebook, Instagram, LinkedIn, Twitter, Viber, and WhatsApp), health services (e.g., general practitioners, psychiatric services), and the community (e.g., Albanian associations, religious and women’s groups, and a television emission on “diaspora TV” for Albanians). Social media recruitment has proven to be an effective recruitment strategy in e-mental health research (Kayrouz et al., 2016; Whitaker et al., 2017), and was used in an RCT with the Arabic and English version of Hap-pas-Hapi, called Step-by-Step, in Lebanon (Cuijpers et al., 2022a; Cuijpers et al., 2022b). For recruitment, an account in Albanian language with the title “Hap-pas-Hapi” was created on Facebook, Instagram, and Twitter. A minimum of three posts per week on these platforms shared study information and illustrations on the program. The social media messages were shared by the accounts of the research team, as well as on pages of Albanian associations, and health institutions that have a connection to Albanian clients and patients. In addition, we posted short movies in Albanian language. In one of them, members of the research team invited people to participate. In another one, the study was explained with the help of animated sketches.

The use of social media influencers, who serve as multipliers of health-related content, is becoming increasingly relevant in digital health communication (Dusberger & Pierau, 2020). A selection of trusted and popular social media influencers in the Albanian community were contacted and a few of them shared information about the present study.

We also compiled a list of Albanian-speaking medical doctors (i.e., family doctors, psychiatrists) and psychologists. We contacted them via phone or visited them in their workplace and asked them to support our recruitment. Flyers and posters were deposited in the waiting rooms at private and public outpatient clinics. A group of psychiatrists installed the app on their Smartphones to show it directly to patients and help them sign up. In addition, they spread the word in their respective networks to support our recruitment.

In addition, we started recruitment efforts within the Albanian communities in Switzerland and in Berlin. A team of a senior researcher, a doctoral student, and several master’s students and interns participated in this process. We organized a series of events with Albanian-speaking associations, where we presented the project and the study and invited people to participate. At these events, the project received positive feedback and interest. One master’s student went to events of Albanian groups and associations, and to the Mosque to distribute flyers and talk to people about the project. Two students at University of Lausanne involved the association of Albanian-speaking students in the French-speaking part of Switzerland. We also hired a series of “cultural brokers” (Wenger, 1998), i.e., members of the community who were offered a small reimbursement for supporting our recruitment by diffusing the information and helping people with the sign-up process. Given the flood of health-related digital content and rampant misinformation during the COVID-19 pandemic, we felt it was important to emphasize that “Hap-pas-Hapi” adheres to evidence-based practices and is a trustworthy intervention. To build trust in the institutions and the research team behind the intervention, we installed a weekly “meet-the-expert” session via Zoom, where people could join us, talk with us, and ask questions about the study and discuss strategies to address mental health in Albanian-speaking communities. Finally, we reduced the number of baseline assessments to reduce dropouts during that phase (see Results section).

When recruitment did not proceed after all these efforts, we sought ethical approval in Austria and Italy to expand the recruitment in those countries. We collaborated with Albanian-speaking health professionals in these countries who promised to support our study.

### Procedure

Interested people accessed the study information and informed consent procedures online, all of which were presented in Albanian language. Applicants were reminded that they were free to decline to participate or withdraw at any time. After giving consent, participants were asked to create an account, first completed the screening questionnaires, and (if screened positive) completed the additional baseline questionnaires. Applicants who were excluded based on one of the exclusion criteria received an on-screen message thanking them for their interest in the study and explaining that they could not participate in this study at this point. People who answered “yes” on the question about imminent risk of suicide received a message saying that it was important that they sought help, providing a list of crisis intervention centres in Switzerland and Germany, along with numbers for telephone counselling in both countries.

Included participants could use the intervention and were invited for post-assessments six weeks after baseline assessments. Three months later, they were invited for follow-up assessments. All measures (pre-, post- and follow-up) were completed online. As soon as the assessment was due, participants saw them as a new “session” on their home screen within the Hap-pas-Hapi programme. If they used the mobile version and had previously agreed to receive automated notifications, they received a pop-up message saying that the assessments were due. E-helpers sent a maximum of three reminders for post- and again for follow-up assessments.

### Randomisation

After sign-up, informed consent and screening, participants were randomly allocated to one of the two treatment conditions and invited to complete the baseline assessments. In the study information, participants learned that they would be randomised into one of two conditions, but we did not provide any further information on the differences between the two versions of Hap-pas-Hapi. Participants were blind to the condition they were allocated to. Randomisation and group allocation (1:1) were done automatically by the system. A permuted block randomisation algorithm (random block lengths of 4, 6, or 8) was used. All assessments were done online, and the study team did not have access to data or randomisation during the trial.

### Sample Size

The power analysis for the current study is described in detail in the protocol paper (see Heim et al., 2020S-b). According to this analysis, we needed 320 participants (completed baseline assessments) to make sure the trial was sufficiently powered.

### Screening Measure

The K10 (Kessler et al., 2002) was used as screening measure. It includes ten items on psychological distress, with a total score ranging from 10 to 50. In line with the STRENGTHS study, we used a score of >15 as an indication of moderate to high levels of psychological distress (de Graaff et al., 2020).

### Primary Outcomes

Questionnaires applied at each assessment time are described in detail in the protocol paper (Heim et al., 2020S-b). The first primary outcome was the Hopkins Symptom Checklist (HSCL-25), which consists of 25 items related to psychological distress (Derogatis et al., 1974).

The second primary outcome was treatment adherence, defined as completing at least three (out of five) sessions. The reason for this definition of treatment adherence was the fact that the main adaptations had been done in the first two sessions. Thus, after session 3, we did not expect group differences with regard to adherence, because the intervention versions were identical. Use of the intervention (i.e., start and completion of sessions, exercises) was automatically registered by the online platform.

### Secondary Outcomes

We used the WHO Disability Assessment Schedule (WHODAS) 2.0 for assessing functioning (Rehm et al., 1999). In addition, we applied the WHO Well-being Index (WHO-5), a 5-item questionnaire measuring current psychological wellbeing and quality of life (Bech et al., 2003). PTSD symptoms were measured using the abbreviated eight-item version of the PTSD Checklist for DSM-5 (PCL-5, Price et al., 2016). And finally, self-defined problems and symptoms were measured using the Psychological Outcome Profiles instrument (PSYCHLOPS, Ashworth et al., 2004).

### Mediators

The German questionnaire «Fragebogen zur Erhebung von Kontrollüberzeugungen zu Krankheit und Gesundheit» (KKG, 'Questionnaire to assess control beliefs about illness and health', Lohaus & Schmitt, 1989) measures three dimensions, i.e., beliefs in internal, social–external, and fatalistic–external illness-related locus of control.

The Illness Perception Questionnaire Revised (IPQ-R, Moss-Morris et al., 2002) measures different kinds of beliefs about an illness (e.g., about its course, consequences, personal control, treatment control, etc.). We only used the second part, which includes assumptions about causes (i.e., personal attributions, risk factors, immunity, accidents, or chance).

Furthermore, we measured working alliance with Hap-pas-Hapi using the Working Alliance Inventory (WAI, Munder et al., 2010) for guided internet interventions (Gómez Penedo et al., 2020).

### Other Measures

We gathered socio-demographic information, including sex, age, marital status, nationality, level of education, employment, and time lived in the host country. And we applied an adapted version of the Client Satisfaction Questionnaire CSQ (Larsen et al., 1979) for internet-based interventions (Boß et al., 2016).

### Statistical Analysis

The original data analysis plan is described in the trial’s protocol paper (Heim et al., 2020S-b). Due to the small sample size (*n* = 97 completed baseline assessments) and high dropout (81%), we only calculated the percentage of users completing at least 3 out of 5 sessions (second primary outcome).

## Results

**Figure 1:**
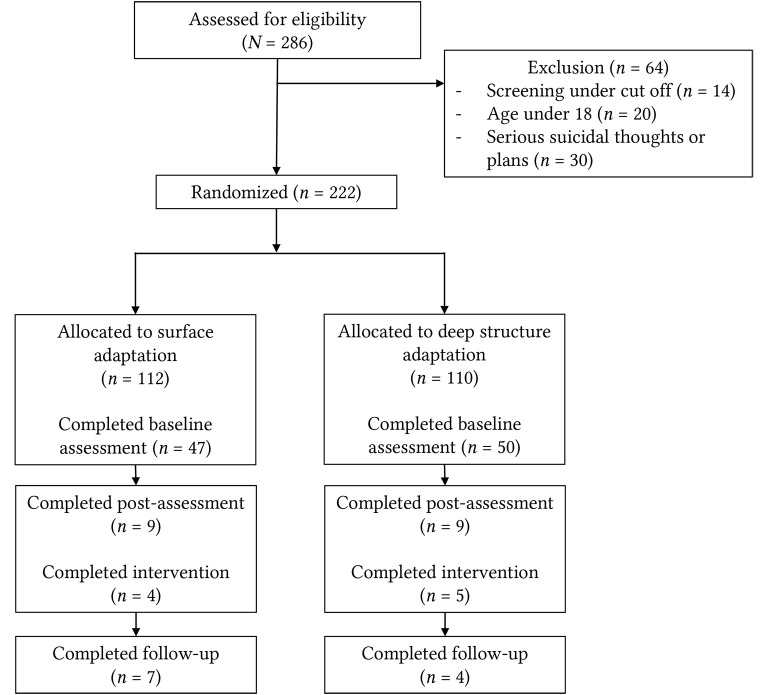
Flow Chart

A total of 222 participants were included in this study (see Figure 1), of which *N* = 112 were assigned to the surface and *N* = 110 were assigned to the deep structure adaptation version of Hap-pas-Hapi. Less than half of participants (*n* = 97, 43.7%) completed baseline assessments.

We first report on the results of the different recruitment strategies. Table 1 shows results of the question “where have you heard about our study”, which was responded by *N* = 145 participants. This table shows that the largest number was recruited through social media (59%), while other strategies such as information events and recruitment through healthcare worker, did not work at all. Our social media posts reached up to 23,000 times, thus we can assume that they were seen. In the first month (i.e., June 2020), 31 people signed up and completed baseline assessments. In the following three months, only 15, 11, and 12 completed baseline assessments. From October 2020, social media posts did no longer result in an immediate increase of participants, although they were seen and shared. From there on, we had between 0 and 6 new participants per month. Influencers’ posts had a short positive effect on recruitment rates. The events in Albanian associations and communities did not lead to an increase in participants, although the project and the application received very positive feedback. Recruitment through healthcare workers did not work at all. In July 2021, the budget for recruitment and for maintaining the platform ran out. Due to very little success of our recruitment strategies, and high drop-out rates (see below), we opted for an early termination of the trial.

**Table 1:** Results of the Question “Where Have You Heard About Study?” (N = 145)

Recruitment channel	*N* (%)^a^
Facebook, Instagram, other social media	86 (59.3%)
Other online platforms	8 (5.5%)
Family member, friends	33 (22.8)
Healthcare worker	1 (0.7%)
Association	7 (4.8%)
Information event	1 (0.7%)
Other	8 (5.5%)

^a^Percentage out of those who responded to the question (*N* = 145).

Descriptive statistics are reported in Table 2. A total of *N* = 145 responded to the socio-demographic questions at the beginning of baseline assessment. The mean age was 30.5 years, with no significant difference between groups (*p* = .338). No significant group differences emerged regarding the other socio-demographic variables.

**Table 2:** Descriptive Statistics (N = 145)

Characteristic	Surface adaptation (*N* = 69) *N* (%)^a^	Deep structure adaptation (*N* = 76) *N* (%)^a^	Total (*N* = 145) *N* (%)^a^	*p* group comparison
**Female gender**	47 (68.1%)	52 (68.4%)	99 (68%)	.969^b^
Age				.338^c^
18-30	47 (68.1%)	49 (64.5%)	96 (66.2%)	
31-40	13 (18.8%)	15 (19.7%)	28 (19.3%)	
41-50	6 (8.7%)	6 (7.9%)	13 (9.0%)	
51-60	3 (4.3%)	5 (6.6%)	8 (5.5%)	
61-70	0	1 (1.3%)	1 (0.7%)	
Nationality				.106^b^
Switzerland	9 (13%)	11 (14.5%)	20 (13.8%)	
Germany	3 (4.3%)	13 (17.1%)	16 (11%)	
Kosovo	32 (46.4%)	31 (40.8%)	63 (43.4%)	
Albania	18 (26.1%)	16 (21.1%)	34 (23.4%)	
Macedonia	3 (4.3%)	2 (2.6%)	5 (3.4%)	
Other	3 (4.3%)	0	3 (2.1%)	
No response	1 (1.4%)	3 (3.9%)	4 (2.8%)	
2nd nationality				.673^b^
None	43 (62.3%)	44 (57.9%)	87 (60%)	
Swiss	4 (5.8%)	10 (13.2%)	14 (9.7%)	
Germany	4 (5.8%)	3 (3.9%)	7 (4.8%)	
Kosovo	9 (13%)	7 (9.2%)	16 (11%)	
Albania	1 (1.4%)	2 (2.6%)	3 (2.1%)	
Macedonia	2 (2.9%)	2 (2.6%)	4 (2.8%)	
Serbia	1 (1.4%)	3 (3.9%)	4 (2.8%)	
Other	4 (5.8%)	2 (2.6%)	6 (4.1%)	
I’d rather not say	1 (1.4%)	1 (1.3%)	2 (1.4%)	
No response	0	2 (2.6%)	2 (1.4%)	
Years lived in host country				.818^b^
Born in Switzerland / Germany	16 (23.2%)	21 (27.6%)	37 (25.5%)	
More than 30 years	2 (2.9%)	4 (5.3%)	6 (4.1%)	
21-30 years	8 (11.6%)	11 (14.5%)	19 (13.1%)	
11-20 years	8 (11.6%)	7 (9.2%)	15 (10.3%)	
5-10 years	16 (23.2%)	12 (15.8%)	28 (19.3%)	
Less than 5 years	19 (27.5%)	21 (27.6%)	40 (27.6%)	
Education				.855^b^
No education	1 (1.4%)	0	1 (0.7%)	
Primary school	0	1 (1.3%)	1 (0.7%)	
Elementary education	3 (4.3%)	3 (3.9%)	6 (4.1%)	
Secondary education	15 (21.7%)	13 (17.1%)	28 (19.3%)	
Technical secondary education	14 (20.3%)	17 (22.4%)	31 (21.4%)	
Undergraduate or BSc degree	20 (29%)	25 (32.9%)	45 (31%)	
Graduate or MSc degree	15 (21.7%)	14 (18.4%)	29 (20%)	
Higher university degree (PhD)	1 (1.4%)	2 (2.6%)	3 (1.4%)	
No response	0	1 (1.3%)	1 (0.7%)	
Marital status				.525^b^
Single	39 (56.5%)	37 (48.7%)	76 (52.4%)	
Married	27 (39.1%)	31 (40.8%)	58 (40.0%)	
Separated	2 (2.9%)	3 (3.9%)	5 (3.4%)	
Divorced	0	2 (2.6%)	2 (1.4%)	
Widowed	1 (1.4%)	3 (3.9%)	4 (2.8%)	
Work status				.100^b^
Paid work	35 (50.7%)	43 (56.6%)	78 (53.8%)	
Non-paid work	4 (5.8%)	6 (7.9%)	10 (6.9%)	
Student	18 (26.1%)	18 (23.7%)	36 (24.8%)	
Retired	1 (1.4%)	2 (2.6%)	3 (2.1%)	
Unemployed (health reasons)	1 (1.4%)	5 (6.6%)	6 (4.1%)	
Unemployed (other reasons)	10 (14.5%)	2 (2.6%)	12 (8.3%)	

^a^Percentage out of those who responded to the question. ^b^*t*-test for independent samples. ^c^Chi-square test.

Drop-out rates were high (see Figure 1). A large percentage of participants (*N* = 125, 56%) was lost already during baseline assessments. In both groups, just nine participants (8%) completed the post-assessments respectively, with no significant group difference (Chi-square test *p* = .968). Post-assessment completion rates out of those who had completed the baseline assessments was 19% for the surface adaptation and 18% for the deep structure adaptation group (Chi-square test *p* = .884). The follow-up assessments were completed by seven (5%) participants from the surface adaptation and four (4%) participants from the deep structure adaptation group.

The number of participants who completed at least three out of five sessions did not differ between the surface and deep structure adaptation group (4.5% and 5.5%, respectively, see Table 3). Due to the large drop-out rate and thus an insufficient number of participants for the planned analyses, we did not continue with further statistical analyses of the questionnaire data.

**Table 3:** Completion Rates

Stage	Surface adaptation (*n* = 112)	Deep structure adaptation (*n* = 110)
Completed baseline	47 (48.5%)	50 (51.5%)
Completed intro	24 (21.4%)	23 (20.9%)
Completed S3	5 (4.5%)	6 (5.5%)
Completed S5	4 (3.6%)	5 (4.5%)

*Note.* intro = introduction; S3 = session 3; S5 = session 5.

## Discussion

In cultural adaptation literature, empirical evidence on different levels of adaptation is lacking, and experimental studies are scarce. The present study aimed to deliver such evidence as a starting point for future studies. For this purpose, two levels of cultural adaptation – surface vs. deep structure (Resnicow et al., 1999) – of an online self-help programme for the treatment of psychological distress were compared in a randomized controlled trial.

Despite an extensive effort through a variety of channels, we were unable to recruit a sufficient number of participants for our trial. Most participants were recruited through social media, while other strategies, such as involving health workers or organising events with Albanian associations, were not successful. This is in line with previous studies (i.e., Harper Shehadeh et al., 2020; Heim et al., 2021). However, the social media recruitment strategy was successful only during the first three months. We can only speculate about the reason for this outcome. It is possible that at start, people who were most motivated for participation enrolled, whereas the large majority could not be convinced with the posts and ads that followed. We conducted a qualitative study (Heim et al., 2024, this issue), showing recruitment materials to a small sample of Albanian-speaking immigrants in Switzerland, to gather feedback and suggestions for future studies.

Upon early termination of the study, we had included a total of 222 participants, of which 204 (92%) were lost to post-assessment (see Figure 1). This large drop-out rate stands in contrast with other Step-by-Step trials. In two RCTs in Lebanon, the drop-out rates (completed post-assessment) were 65% in the Lebanese population (Cuijpers et al., 2022a) and 46% among Syrian refugees (Cuijpers et al., 2022b). It is important to mention that in our study, *N* = 125 (56%) were already lost during baseline assessments and thus did not proceed to the intervention content. One potential explanation for this large drop-out during baseline assessments is the lack of cultural adaptation of the applied questionnaires, and the high stigmatisation of mental health problems in the Albanian-speaking community (Dow & Woolley, 2011; Shala et al., 2020b). However, we used standard measures that are widely used across a large variety of cultural and ethnic groups worldwide. Thus, the questionnaires themselves do not fully explain the large drop-out rates during baseline assessments. It might also be possible that potential participants stopped baseline assessment due to the extensive number of questions, and the related workload. However, the majority (*N* = 80) was lost already during the first questionnaire, i.e., the HSCL. There seem to be other reasons for dropout at this stage.

Against our hypothesis, the drop-out rates did not differ between the two conditions. Our trial was not sufficiently powered to draw meaningful conclusions, but we can at least state that the deep structure adaptation did not lead to a considerable reduction of drop-out compared to the surface adaptation. It is of course also possible that the few participants who continued the intervention were highly motivated in both groups, so that the deep structure adaptation did not make a difference.

Taken together, the low recruitment rate and the large drop-out rate indicate that Hap-pas-Hapi, in its current format, did not meet the needs and expectations of the Albanian-speaking community in Switzerland and Germany. One reason for this result might be the lack of intrinsic motivation to seek help, and a lack of self-efficacy when it comes to one’s help, which is related to fatalistic-external health beliefs (Lohaus & Schmitt, 1989; Shala et al., 2020b). Another reason might be the above-mentioned high stigmatization of mental health problems in the Albanian-speaking community (Dow & Woolley, 2011; Shala et al., 2020b). And yet another reason might be the lack of guidance. In this study, we used a guidance-on-demand model, in which e-helpers do not proactively reach out to participants. Proactively contacting all participants and providing weekly minimal guidance, might have reduced drop-out rates in our study (Torous et al., 2020).

Another important limitation might have been caused by our careful adaptation process itself. We adapted Hap-pas-Hapi to cultural concepts of distress in the Albanian-speaking community (Shala et al., 2020a; Shala et al., 2020b), and the application was only available in Albanian language. However, our ethnopsychological research showed that there are large differences between first- and second-generation immigrants (Pnishi et al., 2024). Second generation immigrants were born and socialized in the host country. Our socio-demographic data shows that 26% of those who had completed the socio-demographic questionnaire were born in Switzerland or Germany. Although we do not know anything about all those who saw our social media posts and did not enroll in the study, we might speculate that a German (or French, for this part of Switzerland) version would have motivated second-generation immigrants to participate in our study. As to the group of first-generation immigrants, a lack of e-health literacy (Norman & Skinner, 2006), and a lack of motivation to use a mobile application for mental health, might have played an important role.

Ethnic minorities are generally under-represented in clinical trials in high-income countries (Hussain-Gambles et al., 2004; Wendler et al., 2006). Our results show that it is not enough to have “good intentions” to include ethnic minorities in research. Without a deep understanding of their concepts and beliefs about health and illness, health services, help-seeking, and research, it may result very difficult to reach them. And despite our extensive ethnopsychological study, careful adaptation, and massive recruitment effort, we seem to have missed key facts about our target population. We can only hope that our post-hoc qualitative study (Heim et al., 2024, this issue) will help us understand the reasons for our difficulties, and gain a deeper understanding of what needs to be done in future studies.

## Supplementary Materials

The Supplementary Materials contain the following items:

- The trial registration (Heim et al., 2020S-a)
- The preregistration for the study (Heim et al., 2020S-b)

HeimE.
BurchertS.
ShalaM.
HoxhaA.
KaufmannM.
Cerga PashojaA.
MorinaN.
SchaubM. P.
KnaevelsrudC.
MaerckerA.
 (2020S-a). Supplementary materials to "Effect of cultural adaptation of a smartphone-based self-help programme on its acceptability and efficacy: Randomized controlled trial"
[Trial registration]. PsychOpen. https://www.clinicaltrials.gov/study/NCT04230135
10.32872/cpe.2743PMC1130391739119053

HeimE.
BurchertS.
ShalaM.
HoxhaA.
KaufmannM.
Cerga PashojaA.
MorinaN.
SchaubM. P.
KnaevelsrudC.
MaerckerA.
 (2020S-b). Supplementary materials to "Effect of cultural adaptation of a smartphone-based self-help programme on its acceptability and efficacy: Randomized controlled trial"
[Preregistration]. PsychOpen. 10.23668/psycharchives.3152
PMC1130391739119053
